# White Blood Cells Classification Using Entropy-Controlled Deep Features Optimization

**DOI:** 10.3390/diagnostics13030352

**Published:** 2023-01-18

**Authors:** Riaz Ahmad, Muhammad Awais, Nabeela Kausar, Tallha Akram

**Affiliations:** 1 Department of Computer Science, Iqra University, Islamabad 44800, Pakistan; 2 Department of Computer Science, COMSATS University Islamabad, Wah Campus, Wah Cantt 47010, Pakistan; 3 Department of Electrical & Computer Engineering, COMSATS University Islamabad, Wah Campus, Wah Cantt 47010, Pakistan

**Keywords:** deep learning, nature-inspired feature selection, leukemia, CNN, white blood cell, classification, medical imaging

## Abstract

White blood cells (WBCs) constitute an essential part of the human immune system. The correct identification of WBC subtypes is critical in the diagnosis of leukemia, a kind of blood cancer defined by the aberrant proliferation of malignant leukocytes in the bone marrow. The traditional approach of classifying WBCs, which involves the visual analysis of blood smear images, is labor-intensive and error-prone. Modern approaches based on deep convolutional neural networks provide significant results for this type of image categorization, but have high processing and implementation costs owing to very large feature sets. This paper presents an improved hybrid approach for efficient WBC subtype classification. First, optimum deep features are extracted from enhanced and segmented WBC images using transfer learning on pre-trained deep neural networks, i.e., DenseNet201 and Darknet53. The serially fused feature vector is then filtered using an entropy-controlled marine predator algorithm (ECMPA). This nature-inspired meta-heuristic optimization algorithm selects the most dominant features while discarding the weak ones. The reduced feature vector is classified with multiple baseline classifiers with various kernel settings. The proposed methodology is validated on a public dataset of 5000 synthetic images that correspond to five different subtypes of WBCs. The system achieves an overall average accuracy of 99.9% with more than 95% reduction in the size of the feature vector. The feature selection algorithm also demonstrates better convergence performance as compared to classical meta-heuristic algorithms. The proposed method also demonstrates a comparable performance with several existing works on WBC classification.

## 1. Introduction

**Figure 1:**
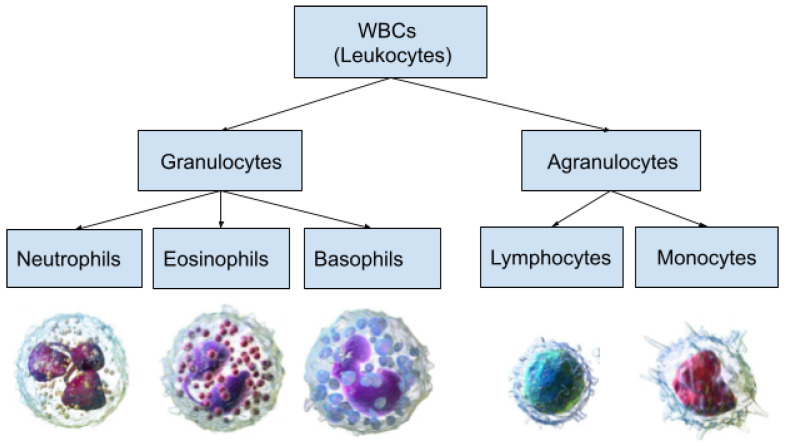
White blood cell subtypes.

Blood is a crucial fluid in the human body that is essential for life. Human blood is made up of plasma and blood cells. Plasma is the yellowish liquid component of blood that is largely water and accounts for 55% of blood volume [1]. The blood also includes proteins, carbohydrates, minerals, hormones, carbon dioxide, and blood cells. Red blood cells (RBCs), white blood cells (WBCs), and platelets (thrombocytes) are the three different types of cellular components found in the blood, each distinguished by their color, texture, and appearance. RBCs, also known as erythrocytes, carry hemoglobin, an iron-containing protein that aids in the delivery of oxygen from the lungs to the tissues. WBCs, also known as leukocytes, are an essential component of the human immune system, assisting the body in fighting infectious diseases and foreign substances [2,3]. Figure 1 demonstrates a classification of WBCs on the basis of their structure. WBCs are primarily of two types, i.e., granulocytes and agranulocytes. The granulocytes have their origin in the bone marrow and are present within the cytoplasm in the form of granules of protein. There are three types of granulocyte cells, namely basophils, eosinophils, and neutrophils. Agranulocytes, which are defined as cells without granules in their cytoplasm, are further divided into two types, i.e., lymphocytes and monocytes [4]. Each type of cell has a unique role in the immune system of the body. For example, neutrophils act as scavengers that surround and destroy bacteria and fungi present in the body. Eosinophils play a role in the general immune and inflammatory responses of the body. An increased level of basophils results in a blood disorder after an allergic reaction. Monocytes fight against infections, remove dead or damaged tissues, and kill cancerous cells; lymphocytes combat bacteria, viruses, and other cells that pose a threat to the body’s ability to function [5]. A detailed analysis of WBCs is very important to assess the overall condition of the human immune system. In particular, WBC analysis is crucial in the diagnosis of leukemia, a type of blood cancer that occurs due to the excessive production of malignant WBCs in the bone marrow. Leukemia diagnosis is performed by one of three main clinical tests, i.e., physical test, complete blood count (CBC) test, and bone marrow test. The first step of CBC is to determine different types of WBCs from the blood samples. This task is mainly performed by hematologists through the visual examination of microscopic images of blood smears. This manual method is labor-intensive, time-consuming, and prone to inaccuracy due to judgment errors influenced by several external factors.

With the recent advancement in digital image processing technology, the automated classification of WBCs using computer vision techniques has attracted significant research interest. However, due to morphological overlap between different subclasses and their structural irregularities, the machine learning-based classification and localization of WBCs is challenging. Deep learning with convolutional neural networks (CNNs) is the most promising method for classification and detection tasks in the field of contemporary medical imaging [6,7]. Despite the fact that CNNs perform best on large datasets, training them takes a lot of data and computational power. The dataset is frequently small and may not be sufficient to train a CNN from scratch. In such a case, transfer learning is frequently used to maximize the effectiveness of CNNs while also decreasing the computational costs [8]. In this approach, the CNN is initially pre-trained on a large dataset consisting of a diverse range of classes and then applied to a specific task [9]. There are various pre-trained neural networks that have won international contests, including VGGNet [10], Resnet [11], Darknet [12], Densenet [13], Mobilenet [14], Inception [15], Xception, [16] etc. Through their capacity for self-learning, these models are able to extract a rich set of features from images that contain substantial semantic information. This helps to achieve a significant level of accuracy for a variety of image classification scenarios. In modern deep learning applications, feature selection is a crucial step which reduces the difficulty of model learning by removing irrelevant or redundant features. The present research is focused on achieving a high level of accuracy with a smaller feature set to reduce the computation costs and memory requirements of expert systems.

The existing works on WBC classification are broadly classified into two categories, i.e., (a) classical methods and (b) deep learning methods. The classical methods consist of approaches which propose efficient preprocessing techniques to extract strong features from WBC images and classify them using baseline classifiers. Some remarkable works in this domain are discussed as follows. In [17], the authors proposed a method which selects the eigenvectors from color images of blood cells based on the minimization of similarities. The Bayesian classifier is then used to classify the eigen cells on the basis of density and color information. In [18], Fuzzy C-means clustering is applied to separate the nucleus and cytoplasm of leukocytes. Then, various geometric, color, and statistical properties are extracted and classified by support vector machines (SVMs). In [19], an image segmentation method is proposed based on mean-shift clustering and boundary removal rules with a gradient vector flow. An ensemble of features is extracted from the segmented nucleus and cytoplasm, which is then classified using a random forest algorithm. In [20], the authors tested the performance of six different machine learning algorithms on 35 different geometric and statistical features. The multinomial logistic regression algorithm outperformed other methods. A stepwise linear discriminant analysis method is proposed in [21], which extracts specific features from blood structure images and classifies them using reversion values such as partial F values. In [22], the authors presented a WBC cancer detection method which combines various morphological, clustering, and image pre-processing steps with random forest classifier. The suggested method uses a decision tree learning method, which uses predictors at each node to make better decisions, in order to categorize various types of cancer.

The second category of works is based on deep learning approaches for WBC classification. The works in this category primarily employ transfer learning of a pretrained deep neural network for feature extraction or classification. Some important works are discussed as follows. In [23], the authors proposed a deep learning method that uses the DenseNet121 [13] model to classify WBC subtypes. The model is optimized with the preprocessing techniques of normalization and data augmentation applied to a Kaggle dataset. The work in [24] first applies a thresholding-based segmentation on the WBC images. Feature extraction from segmented images is performed using VGG16 CNN [10] model learning. The extracted feature vectors are classified using the K-nearest neighbor (KNN) algorithm. In [25], the authors investigated generative adversarial networks (GANs) for data augmentation and employed the DenseNet169 [13] network for WBC classification. In [26], the authors applied Gaussian and median filtering before training the images using multiple deep neural networks. The authors in [27] applied a you-only-look-once (YOLO) algorithm for the detection of blood cells from a smear images. In [28], two techniques are proposed for blood cell identification, namely single-shot multibox detector and an incrementally improved version of YOLO.

Although modern approaches based on transfer learning on deep CNN models achieve a decent level of accuracy for a variety of classification tasks, they all share the use of a large number of features extracted from deep neural networks. This suffers from high computational cost and memory requirements for practical deployment. In most biomedical scenarios, many of these deep features are redundant or contain zeros. Effective dimensionality reduction, or choosing only powerful, discriminant features, increases classifier accuracy while decreasing computational time and expense. WBC classification using deep feature selection is an emerging research area. Few works have reported population-based meta-heuristics for deep feature selection. The authors of [29] have proposed a leukemia detection system in which various features, such as color, texture, shape, and hybrid features, are first extracted from WBC images and then an optimization algorithm inspired by social spiders is used to select the most useful features. In [30], a leukemia detection approach is proposed which combines deep feature extraction using VGGNet and a statistically enhanced salp swarm algorithm for feature selection. Furthermore, the classification of reduced feature vectors was performed using a baseline classifier. The work in [31] proposes a self-designed neural network named W-Net to classify five subtypes of WBCs. The authors also generated a synthetic WBC dataset using a generative adversarial network (GAN).

In this study, we have proposed a hybrid approach for WBCs classification. The proposed approach first creates an ensemble of deep features extracted by applying transfer learning of multiple deep CNNs on WBC images and then performs feature selection using an entropy-controlled nature-inspired algorithm. The main contributions of this work can be summarized in the following steps.

1. Using a synthetic, real-world, large-scale dataset of five WBC types, transfer learning is performed using two deep CNNs, namely Darknet53 and Densenet201, followed by their feature fusion;
2. For feature selection, a nature-inspired meta-heuristic named entropy-controlled marine predators algorithm (ECMPA) is proposed. The proposed algorithm effectively selects only the most dominant features;
3. The reduced feature set is classified using various baseline classifiers with multiple kernel settings;
4. The proposed feature selection algorithm demonstrates a high accuracy with significant reduction in feature size. The algorithm also achieves a better convergence rate as compared to classical population-based selection methods.

The main focus of our manuscript is to present a novel method of deep-feature selection using an entropy-controlled population-based algorithm and show its effectiveness in the domain of WBCs classification for leukemia detection. Since the definition of appropriate image features is a very difficult task due to the morphological similarity of images and subject variability, WBC classification is a pertinent design case for such an approach. The rest of this paper is organized as follows. Section 2 discusses all steps of the proposed WBC classification pipeline, Section 3 presents the results and analysis, and Section 4 concludes the paper.

## 2. Materials and Methods

This section provides a description of all steps of the proposed WBC classification system which are discussed in the following subsections.

### 2.1. Dataset Description

**Figure 2:**
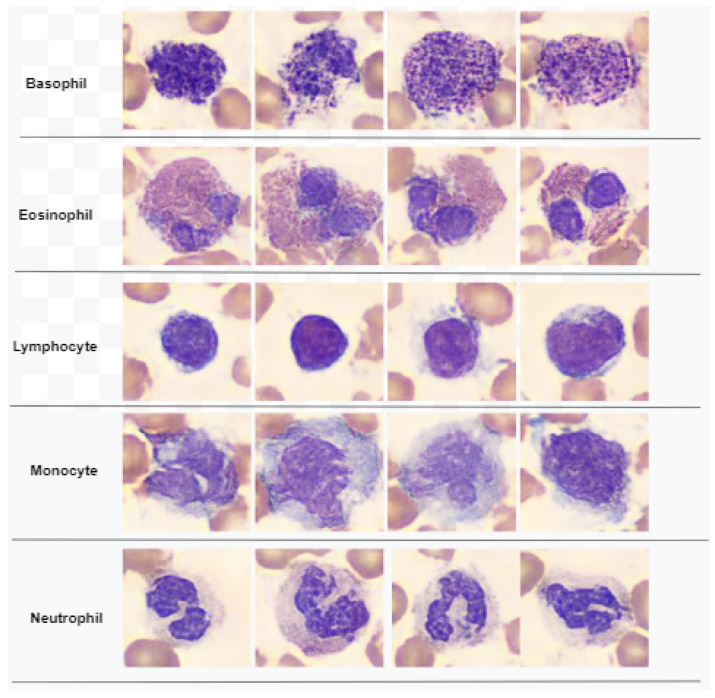
Samplesof WBC images of dataset used in this work.

This work uses the public dataset in [31], which was generated synthetically from a real-world dataset [32] of five sub-types of WBC images, namely neutrophil, eosinophil, basophil, lymphocyte, and monocyte. The synthetic dataset was generated with the help of a deep convolutional generative adversarial network (DCGAN) trained on the original real-world dataset [32] of blood smear images, captured by a Sysmex DI-69 machine and provided by the Catholic University of Korea. The synthetic dataset is composed of 5000 images each of size (128×128×3), with 1000 images belonging for each class. Figure 2 shows samples belonging to all classes of the dataset of [32] used in this work.

### 2.2. WBCs Classification Pipeline

**Figure 3:**
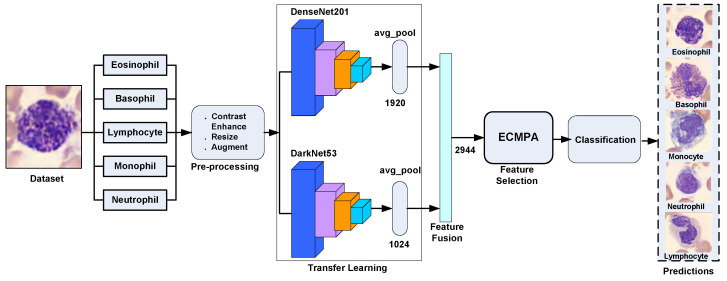
Pipeline of proposed WBCs classification system.

Figure 3 shows the proposed WBCs classification pipeline, whose main computation steps are discussed as follows.

#### 2.2.1. Preprocessing

Image contrast enhancement is a fundamental pre-processing step of many digital image-processing applications. In this work, the input image enhancement is performed with the help of color histogram equalization. The classical method of histogram equalization is applied to grayscale images and performs as redistribution of their intensity. In case of color images, performing histogram equalization on R, G, and B components independently will not necessarily enhance the image. Color histogram equalization can be achieved by converting a color image into a HSV/HSI image and enhancing the intensity while preserving hue and saturation components. The main steps of color histogram equalization are as follows.

- Convert the RGB image into HSI image;
- Obtain the intensity matrix from the HSI image matrix;
- Perform histogram equalization on the intensity matrix;
- Replace the intensity matrix of the HSI image with the histogram-equalized intensity matrix;
- Convert HSI image back to RGB image.

#### 2.2.2. Feature Extraction Using Transfer Learning

The pre-processed image dataset is now subjected to the feature extraction using the transfer learning step. In this work, we performed transfer learning on two well-known deep CNNs, namely DarkNet53 and DenseNet201, which are discussed as follows.

**DarkNet53** is a convolutional neural network proposed as a feature extractor in YOLO3 image detection workflow [12]. It is pretrained on more than a million images from ImageNet database [33]. The pretrained network is able to classify up to 1000 categories of image objects. Details about the various layers in the DarkNet CNN architecture are shown in Table 1. The network has an input layer with a size of 256×256×3 and is primarily made up of convolution layers with sizes of 1×1 and 3×3, totaling 53 layers, including the final fully connected layer but excluding the residual layer. Each convolutional layer is composed of a Conv2d layer followed by a batch normalization (BN) [34] and LeakyReLU [11] layer. The residual layer is added to solve the gradient disappearance or gradient explosion problems in the network [12]. In Darknet53, a significant reduction in parameters is achieved as compared to its previous version, i.e., Darknet19.

In order to perform transfer learning of Darknet53, the last learnable layer of Darknet53, i.e., “Conv5”, is replaced with a new fully connected layer with the number of outputs equal to the number of classes in our WBCs dataset (5 classes). A new softmax layer is created and replaced with the original softmax layer of the network. Similarly, the classification layer of the network is replaced with a new classification layer without class labels. To perform the network training, first the dataset images are resized to 256×256×3 using the nearest neighbor interpolation method, followed by image augmentation which performs random rotation of images in the range of [0,360] degrees and scaling with a factor in the range of [0.5,1]. The deep features are extracted from the “GlobalAvgPool” layer. The DarkNet53 returns a deep feature vector of size 1024 per image.

**DenseNet201**. This deep convolutional neural network is 201 layers deep [13]. It is also pre-trained on Imagenet [33] dataset. DenseNet is designed to overcome the vanishing gradient problem in high-level neural networks. In DenseNet, each layer receives new inputs from all preceding levels and passes on its own feature maps to all following layers. Concatenation is utilized. Each layer receives “collective knowledge” from all preceding levels. This results in a thinner and compact network that achieves a high computational efficiency and memory saving. Table 2 shows the layer details of DenseNet201.

In order to perform transfer learning using DenseNet201, the last learnable layer of the network, i.e., “fc1000” is replaced with a new fully connected layer with 5 classes of the WBCs dataset used in this work. A new softmax layer is created and replaced with the original softmax layer of the network. Similarly, the classification layer of the network is replaced with a new classification layer without class labels. The dataset images are first resized to 224×224 and augmented in a way similar to DarkNet53. From the trained network, the deep features are extracted from the global average pooling layer. DenseNet201 returns a deep feature vector of size 1920 per image. There was no layer freezing carried out during the training process. As a result, the highest possible numbers of trainable parameters, i.e., 18.1 million for DenseNet201 and 41.6 million for DarkNet53, were considered.

#### 2.2.3. Feature Fusion

The deep features extracted from both the Darknet53 and Densenet201 networks discussed above are concatenated together to form a fused feature vector. Let *X* and *Y* be the feature vectors of Darknet53 and Densenet201, respectively, and the fused feature vector *Z* is given as

Z=[XY]

The total size of fused feature vector *Z* is 2944 features per image.

#### 2.2.4. Feature Selection Using Marine Predators Algorithm

Feature selection is an important step which significantly alleviates the curse of dimensionality by reducing the size of the feature vector, selecting only relevant features. The classical feature selection methods based on search algorithms such as complete and sequential search suffer from high computational cost. Population-based meta-heuristic algorithms have been demonstrated as an effective way to solve complex optimization problems [35,36].

The marine predators algorithm (MPA) is a meta-heuristic algorithm that draws inspiration from nature and models the foraging behavior of marine predators (MPs) to find their prey [37]. In the aquatic environment, both the prey and the predator are looking for food at the same time. Position updates for the predator and prey are based on Brownian or Lévy movement, depending on the relative velocities of the two. The goal of MPA, like other swarm optimization techniques, is to choose the best solution (elite) from the population of predators. The MP with the strongest foraging capacity is called elite predator. The MPA is based on the observation that MPs move in Lévy patterns when there are few prey items present and in Brownian patterns when there are many prey items present. Additionally, predators alter their behavior and move to areas with different prey concentrations in the presence of environmental effects. As a result, there are three phases to the position updates in MPA optimization based on the relative velocities of predator and prey: low velocity ratio, unit velocity ratio, and high velocity ratio. The velocity ratio vs. is defined as the ratio between the velocity of prey and predator.

1. In low velocity ratio (v<=0.1), the most suitable movement strategy for the MP is Lévy, whereas the prey moves in Brownian or Lévy movement;
2. In unit velocity ratio (v<=1), if the prey moves in Lévy, the most suitable movement for MP is Brownian;
3. In high velocity ratio (v>1), the best strategy for a predator is not moving at all. In this case, either prey is moving Brownian or Lévy.

**Standard MPA Methodology.** The standard MPA is an iterative, population-based optimization algorithm. The first step is to generate an initial population of solutions. The population matrix of size n×d is generated as follows:

(1) $$P=\left[\begin{array}{cccc} X_{1,1} & X_{1,2} & \cdots & X_{1,d}\\ X_{2,1} & X_{2,2} & \cdots & X_{2,d}\\ \vdots & \vdots & \vdots & \vdots \\ X_{n,1} & X_{n,2} & \cdots & X_{n,d} \end{array}\right]$$

where *n* is the size of population, i.e., number of search agents (each predator and prey are searching for food and can be considered as a search agent), and *d* is the dimension (no. of variables) of each agent. Each variable of initial solution is uniformly distributed over the search space computed as

(2) $$X_{i}=l_{b}+rand\times \left(u_{b}-l_{b}\right)$$

where $l_{b}$ denotes the lower bound, $u_{b}$ denotes the upper bound, and rand is a uniformly distributed random number from the interval [0,1]. Based on the concept of survival of the fittest, the top predators have the best foraging capabilities. Therefore, the fittest solution is nominated as the best predator and used to construct a matrix called *Elite*. In an iteration *I*, the Elite matrix is constructed as

(3) $$Elite=\left[\begin{array}{cccc} X_{1,1}^{I} & X_{1,2}^{I} & \cdots & X_{1,d}^{I}\\ X_{2,1}^{I} & X_{2,2}^{I} & \cdots & X_{2,d}^{I}\\ \vdots & \vdots & \vdots & \vdots \\ X_{n,1}^{I} & X_{n,2}^{I} & \cdots & X_{n,d}^{I} \end{array}\right]=\left[\begin{array}{c} \bar{X}\\ \bar{X}\\ \vdots \\ \bar{X} \end{array}\right]$$

where $\bar{X}$ is the top predator vector which is replicated *n* times to construct the Elite matrix. At the end of each iteration, the Elite matrix will be updated if the fittest predator of a population is replaced by another better predator. Another matrix named Prey is generated with the same dimensions as Elite. The Prey matrix is computed as

(4) $$Prey=\left[\begin{array}{cccc} X_{1,1} & X_{1,2} & \cdots & X_{1,d}\\ X_{2,1} & X_{2,2} & \cdots & X_{2,d}\\ \vdots & \vdots & \vdots & \vdots \\ X_{n,1} & X_{n,2} & \cdots & X_{n,d} \end{array}\right]$$

where $X_{i,j}$ denotes the *j*th dimension of *i*th prey. During first iteration, the Prey matrix is equal to randomly generated population matrix P. In all subsequent iterations, the Prey is updated and its values are used to compute the Elite matrix. The update of the Prey matrix is carried separately in three phases of MPA optimization. These phases include:

**Phase 1:** This phase corresponds to the high velocity ratio and happens in the first ${\left(\frac{1}{3}\right)}^{rd}$ of maximum iterations of algorithm where exploration is more significant. The update rule of this phase is given as:

(5) $$\begin{array}{c} \bar{Stepsize_{i}}=\bar{R_{B}}⨂\left(\bar{Elite_{i}}-\bar{R_{B}}⨂\bar{Prey_{i}}\right),\;\forall i=1,\cdots ,n \end{array}$$

(6) $$\begin{array}{c} \bar{Prey_{i}}=\bar{Prey_{i}}+P.\bar{R}⨂\bar{Stepsize_{i}},\;\forall i=1,\cdots ,n \end{array}$$

where $\bar{Prey_{i}}$ is a vector of Prey matrix, $\bar{R_{B}}$ and $\bar{R}$ are vectors of dimensions d containing random numbers from Normal and Uniform distribution, respectively, *P* is constant value equal to 0.5, and ⨂ shows element-wise multiplication.

**Phase 2:** This phase corresponds to unit velocity ratio when predator and prey are moving at the same pace. This is the phase which occurs for intermediate ${\left(\frac{1}{3}\right)}^{rd}$ of iterations, where exploration and exploitation matters. The update rules for this phase are given as follows:

(7) $$\begin{array}{c} \bar{Stepsize_{i}}=\bar{R_{L}}⨂\left(\bar{Elite_{i}}-\bar{R_{L}}⨂\bar{Prey_{i}}\right),\;\forall i=1,\cdots ,\frac{n}{2} \end{array}$$

(8) $$\begin{array}{c} \bar{Prey_{i}}=\bar{Prey_{i}}+P\bar{R}⨂\bar{Stepsize_{i}},\;\forall i=1\cdots ,\frac{n}{2} \end{array}$$

(9) $$\begin{array}{c} \bar{Stepsize_{i}}=\bar{R_{B}}⨂\left(\bar{Elite_{i}}-\bar{R_{B}}⨂\bar{Prey_{i}}\right),\;\forall i=\frac{n}{2}+1,\cdots ,n \end{array}$$

(10) $$\begin{array}{c} \bar{Prey_{i}}=\bar{Elite_{i}}+P.CF⨂\bar{Stepsize_{i}},\;\forall i=\frac{n}{2}+1\cdots ,n \end{array}$$

(11) 

where $\bar{R_{L}}$ is vector of size d containing random numbers based on Lévy distribution, $CF={\left(1-\frac{I}{I_{Max}}\right)}^{\left(2\frac{I}{I_{Max}}\right)}$ is an adaptive parameter to control the step size for predator movement, *I* is the current iteration, and $I_{Max}$ is the maximum number of iterations.

**Phase 3:** This phase corresponds to low velocity ratio when predator is moving faster than prey. This scenario happens in the last ${\left(\frac{1}{3}\right)}^{rd}$ iterations of the optimization phase where exploitation matters. The update rules for this phase are given as follows:

(12) $$\begin{array}{c} \bar{Stepsize_{i}}=\bar{R_{L}}⨂\left(\bar{Elite_{i}}-\bar{Prey_{i}}\right),\;\forall i=1,\cdots ,n \end{array}$$

(13) $$\begin{array}{c} \bar{Prey_{i}}=\bar{Elite_{i}}+P.CF⨂\bar{Stepsize_{i}},\;\forall i=1\cdots ,n \end{array}$$

The next step is to model the behavioral change in MPs as a result of environmental effects. These effects are known as fish aggregating devices (FADs). The FADs are known as local optima; therefore, the prey and predators must perform longer jumps during simulation to avoid stagnation in local optima. The update of Prey matrix considering the FAD effect is mathematically represented as follows:

(14) $$\bar{Prey_{i}}=\left\{\begin{array}{ccc} \bar{Prey_{i}}+CF\left[\bar{X_{min}}+\bar{R}⨂\left(\bar{X_{max}}-\bar{X_{min}}\right)\right]⨂\bar{U} & \; & r\leq FADs\\ \bar{Prey_{i}}+\left[FADs\left(1-r\right)+r\right]\left(\bar{Prey_{r1}}-\bar{Prey_{r2}}\right) & \; & r>FADs) \end{array}\right\}$$

where FADs=0.2 is the probability of occurrence of FAD effects, $\bar{U}$ is a randomly generated binary vector, *r* is the uniform number in [0,1], $X_{max}$ and $X_{min}$ are the vectors containing lower and upper bounds of dimensions, respectively, and r1, r2 are the random indices of the Prey matrix.

After the Prey matrix is updated using Equations (6)–(13), and incorporating the FAD effects of Equation (14), this matrix is evaluated for fitness function. The fitness of each solution of current iteration is compared to its equivalent solution in prior iteration. If the current solution is more fitted, it replaces the previous one. In the next iteration, the best solution of Prey is used to generate the Elite matrix and update the Prey matrix using Equations (6)–(13).

#### 2.2.5. Entropy-Controlled MPA for Feature Selection

In this work, we proposed a multi-level feature selection algorithm named entropy-controlled marine predators algorithm (ECMPA). The proposed technique is based on two stages of feature selection, the first of which corresponds to feature reduction based on the entropy of the fused feature vector, followed by additional feature reduction based on the MPA. The main computational steps of ECMPA are discussed in Algorithm 1.

**Notations:** In Algorithm 1, matrices and vectors are represented as double struck characters (e.g., F) and scalars are represented as normal letters.

The algorithm receives as inputs the fused feature matrix F, the label vector L, the entropy-controlled feature reduction parameter $e_{c}$, the maximum number of iterations $I_{Max}$, and the population size *N*. Other constant parameters specific to MPA are upper bound $u_{b}$, lower bound $l_{b}$, threshold *t*, constant *P*, Levy coefficient β, and fish aggregating devices effect $F_{ADS}$. The matrix F is of dimensions $n_{t}\times D$, where $n_{t}$ is the number of training images and *D* is the number of features extracted from the feature fusion step. The first level of feature extraction, i.e., entropy-based, is performed by steps 4–7. Step 4 computes the entropy of each column of F and returns a vector E of size 1×D. Step 5 sorts E in descending order, thus returning the sorted vector $E_{2}$ along with indices stored in I. Step 6 extracts the indices of the first $e_{c}$ percentage of features with maximum entropy. Step 7 extracts features in these indices from F and stores them in $F_{2}$. The task for generating initial population matrix P using Equation (1) is performed in Steps 11–15. Step 13 corresponds to Equation (2), where rand() computes a random number from uniform distribution from interval [0,1]. In Steps 19–26, the fitness function of each individual of P is computed using CostFunction and stored in fitness vector Fit. The best fitness is stored in $fit_{g}$ and best individual is stored in $x_{gb}$. The Elite matrix E is computed in Step 28, by performing the Repeat function which concatenates *N* copies of $x_{gb}$ along the first dimension (i.e., column-wise). Steps 31–37 perform update of Prey matrix P according to Phase 1 (Equations (5) and (6)). In Step 32, the function randn(1,D) returns a vector of size 1×D, containing random numbers from Normal distribution. The Phase 2 of MPA according to update rules of Equations (7)–(11) is performed by Steps 39–57. In Step 43, Levy(β,D) generates a vector of size *D* containing random numbers from Lévy Distribution. Steps 59-66 perform the P matrix update according to Equations (12) and (13) of Phase 3. In Steps 68–83, the FAD effects are added to the Prey according to update rules of Equation (14). In each iteration, Steps 85–92 correspond to the memory-saving step where the updated Prey matrix *P* is evaluated for CostFunction and best individual of $X_{gb}$ is obtained. Steps 95–97 correspond to the output step where $S_{F}$, i.e., a binary vector of size *D* is obtained. The indices of non-zero entries of $S_{F}$ correspond to the indices of selected features of the fused feature vector.

In Steps 100–115 the execution of CostFunction is demonstrated, which performs the computation of fitness value of each individual solution. The function accepts as inputs the entropy-reduced feature vector $F_{2}$, the label vector L, and a binary vector a computed by comparing the entries of *i*th solution of P with the threshold *t*. The Step 104 extracts all the features of $F_{2}$ whose indices correspond to non-zero values of a, and then the function partition splits the feature matrix $F_{2}$ and label vector L into training feature set $F_{train}$, testing feature set $F_{test}$, training label set $L_{train}$, and testing label set $L_{test}$ with a splitting ratio $h_{o}$. In the subsequent steps of the function, model training and prediction is performed using KNN classifier and fitness value (cost) is computed using the classification error metric. An individual with a smaller cost value is the fitter individual.
**Algorithm 1:** ECMPA for feature selection.
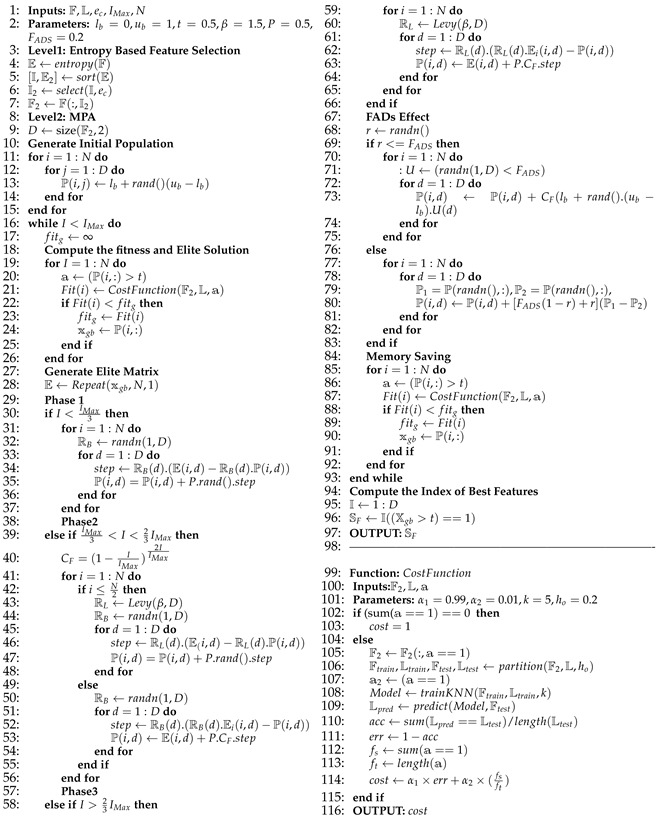


#### 2.2.6. Classification

The indices of the reduced feature set extracted from the ECMPA step discussed above are used to perform training and validation using baseline classifiers with multiple feature settings. In this work, we used KNN [38] and SVM [39] classifiers with multiple kernel settings.

## 3. Results and Discussion

**Figure 4:**
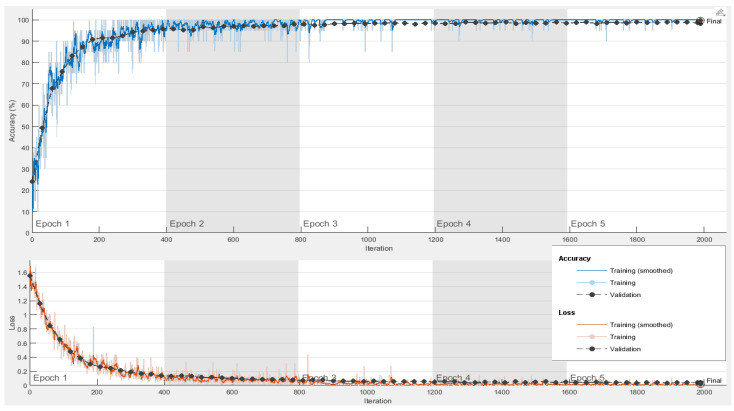
Training accuracy and loss function plots for DenseNet201 network.

**Figure 5:**
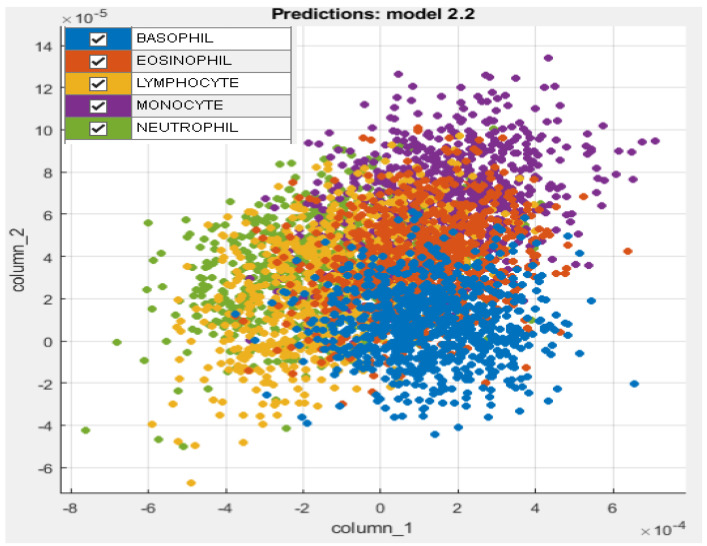
Extracted deep features from proposed ECMPA.

**Figure 6:**
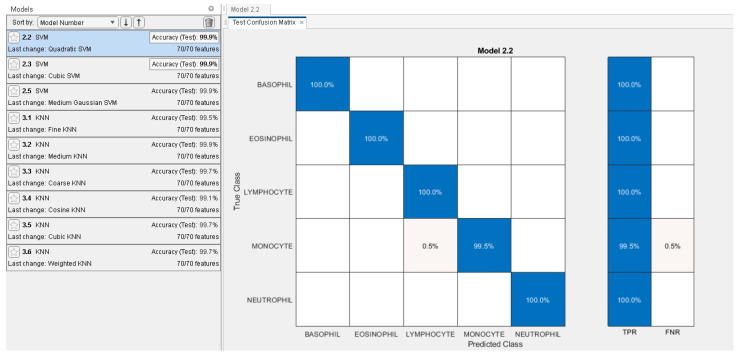
Classification results of proposed WBCs classification system. Left: Test accuracy achieved by SVM and KNN classifiers with several kernels. Right: Confusion matrix of SVM with quadratic kernel.

The proposed system was implemented in Matlab 2021 on Windows 10 64-bit using a Core i5, 2.5 GHz CPU, and 8 GB of RAM. The dataset of 5000 thousand images was split into training and testing with an 80% splitting ratio in order to perform transfer learning using DenseNet201 and DarkNet53 deep models. Table 3 demonstrates the main model training parameters for deep transfer learning. For both networks, a significant level of training and validation accuracy was achieved with five epochs. Figure 4 shows the accuracy and loss function plots for DenseNet201. In order to extract the indices of the most dominant features, the fused feature vector of size 2944 features was then subjected to feature reduction using ECMPA. The reduced set of features is then used to train the KNN and SVM classifiers with multiple kernel settings. In order to perform the classification task, testing images are applied to the trained deep models and a fused feature vector is obtained. The reduced feature vector is generated by using the indices obtained by the ECMPA. This is then classified using the trained KNN and SVM classifiers. Figure 5 demonstrates a set of reduced features extracted from the ECMPA step. Figure 6 demonstrates the results of the proposed WBCs classification system with various kernels of SVM and KNN classifiers. The SVM classifier achieves a 99.9% accuracy with a reduced feature set consisting of 70 strong features. The confusion matrix of SVM with the quadratic kernel is also demonstrated. The high value of true positive rate (TPR) and low value of false negative rate (FNR) are achieved for all image classes.

**Figure 7:**
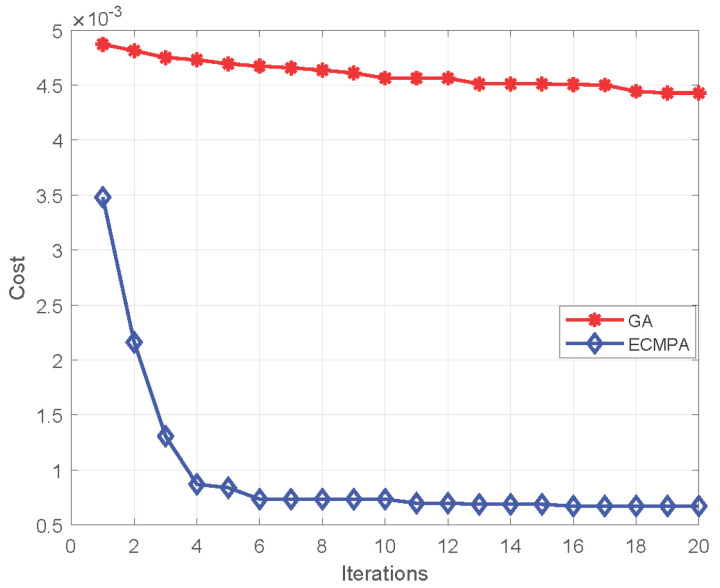
Convergence performance of ECMPA and GA.

In Figure 7, the convergence of the proposed ECMPA is compared with a classical population-based meta-heuristic algorithm, i.e., genetic algorithm (GA). The graph demonstrates that ECMPA achieves a better value of cost function with a smaller number of iterations.

Table 4 shows an accuracy comparison of the proposed approach with some recent works on WBC classification using deep learning networks that use similar datasets. The proposed method shows a comparable or even better accuracy performance with a smaller number of features as compared to other works. This demonstrates the validity of the proposed approach.

### Statistical Significance

Obtaining a certain level of confidence in the proposed strategy is the main goal of this statistical investigation. We use the analysis of variance (ANOVA) [44] to compare the means of several distributions in order to determine whether the results are statistically significant. We consider classification accuracy as a performance characteristic for our proposed framework. In order to implement ANOVA, we performed a series of tests to validate the assumption of normality using the Shapiro–Wilk test [45], and homogeneity of variance using the Bartlett’s test [44]. In these testing procedures, we used 1% level of significance (i.e., α=0.01). The means of classification accuracy values for the selected classifiers, i.e., SVM, KNN, and NNN, as $\mu_{1},\mu_{2}$, and $\mu_{3}$, respectively. For each of the tests mentioned above, the null hypotheses are considered true if the computed Shapiro–Wilk *p*-values are less than or equal to α; otherwise, the alternative hypotheses are affirmed as true. The *p*-values of SVM, KNN, and NNN classifiers are obtained as $p_{1}=0.784,p_{2}=0.7124$, and $p_{3}=0.7031$, respectively. The Chi-squared probability from the Bartlett’s test is $p_{ch}=0.85$. From these *p*-values, we fail to renounce the null hypotheses and confidently claim that our accuracy data are normally distributed with homogeneous variances.

Table 5 presents the statistical results obtained from the ANOVA test including the sum of squared deviation (SS), degree of freedom (df), F-statistics, mean squared error (MSE), and *p*-value. The obtained *p*-value is 0.705, which is greater than α and leads to the conclusion that the means of three classifiers are identical.

**Figure 8:**
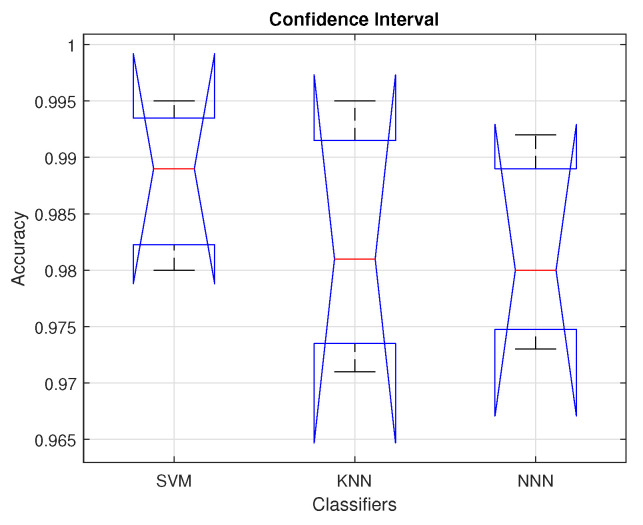
Confidence interval for selected classifiers.

Figure 8 shows the confidence interval plots of accuracy values of the three selected classifiers. In the figure, red bars present the average accuracy, whereas the black bars present the 99% confidence limits of each classifier. In addition, the blue bars show lower and upper quantile points obtained by performing the above-mentioned statistical tests. From the figure, we can observe that the SVM classifier achieves a higher average accuracy with relatively smaller confidence interval size as compared to other classifiers. The quantile points of each classifier lie within their respective confidence limits. The higher *p*-values resulting from these quantile points lead to the acceptance of null hypotheses, which means significant differences in the accuracy distribution of the classifiers.

## 4. Conclusions

WBCs classification is a vital step in the correct diagnosis of Leukemia. The existing manual methods of WBC classification are labor-intensive and error-prone. Automated WBC classification using computer vision techniques is an emerging paradigm. Modern approaches using deep neural networks achieve a significant level of accuracy for a variety of tasks. However, these neural networks suffer from exorbitant computational complexity, processing power, and memory requirement owing to very large feature sets. Therefore, an efficient feature reduction is essential to make deep neural networks feasible for real-time biomedical applications. This work proposes a complete WBCs classification pipeline that performs transfer learning using deep neural networks followed by an efficient feature reduction algorithm. The proposed feature reduction method is validated using several baseline classifiers with multiple kernel settings. An accuracy of 99.9% is achieved with a feature reduction of 95%, which demonstrates the feasibility of the proposed WBCs classification method. While the proposed approach has been applied to an augmented clean dataset containing only WBC subtype images, the ECMPA feature selection algorithm can be applied in any blood cell classification setup with little tuning of parameters. In the future, we plan to extend this work to a more challenging dataset for clinical-grade classification of other cell entities such as platelets and red blood cells, among others. The proposed algorithm can also be tested on the bench mark datasets for other diseases such as skin lesion and brain tumors, among others. In order to address the “*curse of dimensionality*”, other similar bio-inspired meta-heuristics can be investigated to obtain a trade-off between classification accuracy and computational complexity. 

## Figures and Tables

**Table 1:** DarkNet53 layer architecture.

Layer Type	Filters	Filter Size	Stride Size	Repeat	Output Size
Input	-	-	-	-	224×256
Convolutional	32	3×3	1	1	256×256
Convolutional	64	3×3	2	1	128×128
Convolutional	32	1×1	1	1	
Convolutional	64	3×3	1		
Residual					128×128
Convolutional	128	3×3	2	1	64×64
Convolutional	64	1×1	1	2	
Convolutional	128	3×3	1		
Residual					64×64
Convolutional	256	3×3	2	1	32×32
Convolutional	128	1×1	1	8	
Convolutional	256	3×3	1		
Residual					32×32
Convolutional	512	3×3	2	1	16×16
Convolutional	256	1×1	1	8	
Convolutional	512	3×3	1		
Residual					16×16
Convolutional	1024	3×3	2	1	8×8
Convolutional	512	1×1	1	4	
Convolutional	1024	3×3	1		
Residual					8×8
GlobalAvgPool					
Fully Connected	1000				
Softmax					

**Table 2:** DenseNet201 layer architecture.

Layer Type	Composition	Repeat	OutSize
Input	–	–	224×224
Convolution	Conv(7×7), stride 2		112×112
MaxPool	(3×3), stride 2		56×56
Dense Block 1	Conv(1×1)	6	
	Conv(3×3 )		56×56
Transition Layer 1	Conv(1×1)	1	56×56
	Avg Pool(2×2), Stride 2		28×28
Dense Block 2	Conv(1×1)	12	
	Conv(3×3 )		28×28
Transition Layer 2	Conv(1×1)	1	28×28
	Avg Pool(2×2), Stride 2		14×14
Dense Block 3	Conv(1×1)	48	
	Conv(3×3 )		14×14
Transition Layer 3	Conv(1×1)	1	14×14
	Avg Pool(2×2), Stride 2		7×7
Dense Block 4	Conv(1×1)	32	
	Conv(3×3 )		7×7
Classification Layer	7×7 Global Avg. Pool		
	1000D fully Connected, softmax		1×1

**Table 3:** Model training parameters for transfer learning of DenseNet201 and DarkNet53 models.

Property	Value	Property	Value
Kernel	sdgm	Initial Learning Rate	1× $10^{-4}$
Execution Environment	Auto	MiniBatch Size	20
MaxEpochs	5	Validation Frequency	30
Dropout rate	0.1	Stride Size	1

**Table 4:** Performance Comparison of proposed method with some existing works. ×: Not done, N.A: Information not available.

Work	Deep Learning Model	Feature Selection	Feature Vector Size	Classifier	Accuracy %
[40]	GoogleNet, ResNet-50	Maximal Information Coefficient, Ridge Regression Model	755	Quadratic Discriminant Analysis	97.95
[41]	AlexNet	×	1000	CNN	98.4
[42]	PatternNet fused ensemble of CNNs	×	N.A	CNN	99.90
[43]	ResNet and Inception	Hierarchical Approach	N.A	ResNet and Inception	99.84
**This Work**	**DenseNet201 and DartkNet53**	**ECMPA**	**76**	**SVM and KNN**	**99.6**

**Table 5:** Statistical test results based on ANOVA using accuracy metric.

V-Source	SS	df	MSE	F-Statistics	*p*-Value
Between	7.0812 $\times 10^{-5}$	2	3.6333 $\times 10^{-5}$	0.37	0.705
Within	5.9123 $\times 10^{-4}$	6	9.8222 $\times 10^{-5}$	-	-
Total	6.6232 $\times 10^{-4}$	8	-	-	-

## Data Availability

The data for this work shall be available upon request.

## Abbreviations

The following abbreviations are used in this manuscript:

WBC	White blood cell
CNN	Convolutional neural network
DNN	Deep neural network
SVMs	Support vector machines
MPA	Marine predators algorithm
ECMPA	Entropy-controlled marine predators algorithm
KNN	K-nearest neighbors
TPR	True positive rate
FNR	False negative rate
GAN	Generative adversarial network
GVF	Gradient vector flow
